# Understanding the Key Factors of Older Adults’ Continuance Intention in Congregate Meal Halls

**DOI:** 10.3390/foods10112638

**Published:** 2021-10-30

**Authors:** Wang-Chin Tsai, Xuqi Chen

**Affiliations:** 1 Department of Creative Design, National Yunlin University of Science & Technology, Yunlin 640, Taiwan; wangwang@yuntech.edu.tw; 2 Department of Agricultural and Resource Economics, University of Tennessee, Knoxville, TN 37996, USA

**Keywords:** elderly, congregate meal, trust, continued intention

## Abstract

Eating congregate/community meals with friends promotes a balanced and healthy diet among older adults. It is helpful for postponing aging, preventing chronic diseases, and improving their quality of life. However, little research has examined the continuance intention for older adults with the congregate meal program in Taiwan. This study established a model for key factors of older adults’ continuance intention dining at senior meal halls, and hypotheses to explain them, and subsequently designed questionnaires and scales. By analyzing the longitudinal data collected from 416 individuals using survey questionnaires, we found that the perceived service quality is the main factor that affects the perceived satisfaction, and the perceived satisfaction of the older adults plays an important role in this survey. It showed that if the older adults are satisfied with the service quality provided by the senior meal halls, which will accordingly affect the post-use trust, they will show a positive continuance intention to participate in the senior meal halls. We also found that the older adults have positive views on the planning and service contents of the existing senior meal halls. Together, these results illustrate the process and provide comprehensive insights and evidence to create a better user experience and improve the satisfaction of the congregate meal for older adults.

## 1. Introduction

**Figure 1:**
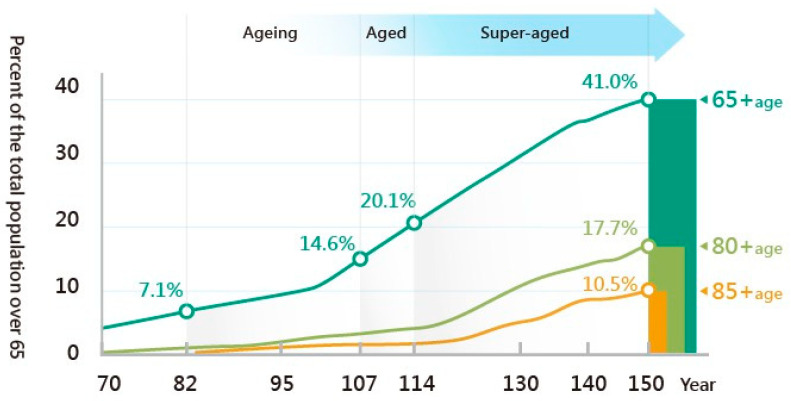
Aging process of Taiwan [2].

As defined by the World Health Organization (WHO), a society with 20% of the total population aged over 65 years old is classified as a super-aged society [1]. According to the statistics of the Department of Household Registration, Ministry of the Interior, Taiwan is expected to enter the super-aged society in 2025, meaning that 20% of the population will be over 65 years old [2] (Figure 1), which would imply a severe aging population structure in Taiwan. Retired older adults may lose their goals in life, and may feel insignificant [3]. Additionally, when older adults lose their goals in life, consequent brain and physiology degeneration may occur, which can lead to serious social problems, such as dementia accelerated by loneliness [4], suicide due to depression [5], and even dying alone. Since these issues involve enormous consumption of medical and social resources, the current situation presents a challenge for the successful aging of older adults [6].

**Figure 2:**
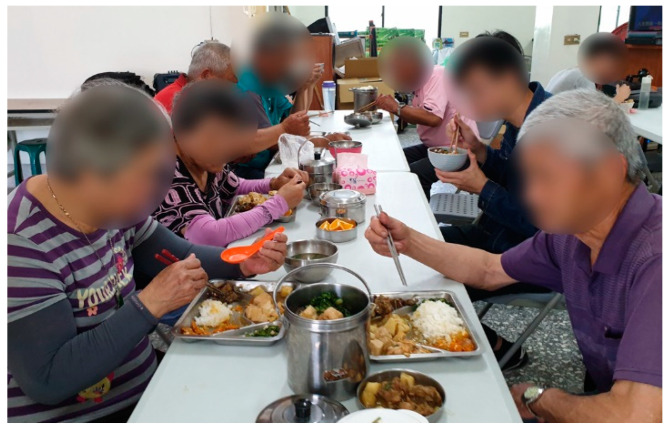
Older adults eat in the senior meal hall. (The image was taken by the author).

In previous decades, there has been a growing emphasis on “aging in place” globally [7], hoping to call more attention to the health, residential environment, psychology, diet, and interpersonal relationships of older adults [8]. Social Eating Programs for older adults have mentioned the nutritional risks and social isolation of older adults living in communities. According to Fulkerson [9], using Meals on Wheels, Eating with Friends, and Congregate/Community Meals can promote a balanced and healthy diet among older adults, which is helpful for postponing aging, preventing chronic diseases, and improving their quality of life [10]. Meanwhile, these programs can also alleviate problems, such as inconvenience in preparing meals, loneliness and depression, and a lack of activities. The Ministry of the Interior (Taiwan) has listed congregate/community meals in Welfare Law concerning older adults since 1997. For communities near rural areas, the establishment of congregate/community meals and senior meal halls is essential, due to the severe aging population structure and migration of young and middle-aged populations [11]. In addition, although senior meal halls have achieved initial success, there are still a considerable number of older adults who feel insecure about local aging and senior meal halls, which even lead to distrust, rejection, aversion [12], and reluctance in dining out. Taking Yunlin County in this study as an example, where the seniors account for the second-highest percentage of the population of Taiwan’s 22 cities and counties, the county government has subsidized the establishment of several senior meal halls, where nearly 8000 people eat every day. This fact indicates that the older adults in Yunlin County have a certain demand for catering services. Therefore, it is necessary to look for experiences from the older adults who have dined at senior congregate meal halls, understand their pre-use trust, post-use trust, satisfaction, and continuance intention, through which we can better understand their experience, feelings, and mental processes when experiencing these meal halls (Figure 2). Our results can contribute to their specification by providing information about the reasons that drive the factors of this model.

This study established a model for key factors of older adults’ continuance intention dining at senior meal halls, and hypotheses to explain them. From a questionnaire formed from scales used in previous studies, data was gathered to enable structural equation modeling to be conducted to test these hypotheses. The findings allow suggestions to be put forth in order to shape aging policy to guide practitioners, and to support government decision-making.

## 2. Theoretical Framework and Research Hypotheses

### 2.1. Trust

As one of the factors explaining consumers’ attitude, satisfaction, commitment, or behavioral intention, trust plays a significant role in uncertain and risky circumstances [13,14]. Generally, trust is a mental state interpreted as positive results of “perceived probabilities”, “confidence”, or “expectations” [15]. Trust is a dynamic concept [16] that changes, develops, and accumulates through repeated behavior [17]. The trust level of consumers will change through use or experience, and there is a feedback cycle of “trust-action-study-trust”, which may repeatedly run [18]. Therefore, trust can be classified as pre-use trust and post-use trust, by which to describe differences of consumers’ trust in products or services through potential influencing factors, and understand the value and influence of products or services on consumers [19]. While the social participation of older adults and the environment complement each other, trust is the basis of social participation [20]. Therefore, to improve older adults’ participation level in the senior meal halls, and understand its influence on older adults, it is necessary to understand the difference between pre-use trust and post-use trust. Older adults’ welfare and quality of life could be focused on two aspects: food, and service. In this study, pre-use trust represented the trust level before the older adults participated in community senior meal halls, while post-use trust represented the trust level after the older adults had participated in the community senior meal halls. Pre-use trust is the prerequisite and influencing factor of post-use trust [16]. That is, in addition to the relevant influencing factors of the topic, post-use trust can also be affected by pre-use trust. Therefore, the hypothesis is as follows:

**Hypothesis** **1** **(H1).** *There is a significant positive correlation between pre-use trust and post-use trust in older adults’ participation in senior meal halls.*

### 2.2. Perceived Risk (PR) and Perceived Value (PV)

Initially belonging to psychology, perceived risk refers to the expected negative effects that consumers feel when they buy specific products [21]. Due to factors such as retirement, the decline of physical functions, and social and economic status, older adults are likely to have social-psychological pressure, leading to a higher risk of depression [22]. In comparison with others, older adults are more likely to feel insecure. Although senior meal halls have been implemented for many years in Taiwan, many people still do not know much about them. Therefore, older adults may have concerns about issues such as food security [23] and economic cost [24]. The community service of senior meal halls mainly includes two aspects of “food” and “service”. “Food” belongs to the physical health dimension, which also includes the economic dimension, while “service” belongs to the psychological dimension [25]. In this study, perceived risk mainly explored the practical problems that older adults might face before or when participating in senior meal halls. Meanwhile, trust is one of the determinants to reduce perceived risk and uncertainty [26]. The higher the trust of older adults in senior meal halls, the lower the expected perceived risks are when they participate in community senior meal halls. Therefore, the hypothesis is as follows:

**Hypothesis** **2** **(H2).** *There is a significant negative correlation between pre-use trust and perceived risk in older adults’ participation in senior meal halls.*

Value is the reflection of a capital cost optimization related to purchasing or production, which creates high-performing (value in use) and attractive (noble value) products or services for consumers [27]. On the other hand, perceived value is the value of products or services as perceived by consumers, which can be seen as the trade-off between perceived benefits and perceived costs [28]. Consumers will repeatedly patronize companies or enterprises with higher perceived value [29]. Although there is usually a negative correlation between perceived value and perceived risk [30], the perceived risk of this study focused more on reality, while the perceived value focused more on psychology. Therefore, no discussion will be made concerning the two factors. Mentally speaking, with certain accessibility, senior meal halls have lower participation requirements and offer opportunities to interact with others, which is one of the positive ways in which to achieve successful aging [31]. Therefore, taking senior meal halls as a platform and medium promotes older adults maintaining their physical and mental health, while older adults can encourage and support each other, and chat and interact to expand their social network [32]. Furthermore, the improvement of trust can also promote the improvement of perceived value [33]. Thus, the higher the trust in senior meal halls among older adults, the higher the probability of a positive perception of the community senior meal halls. Therefore, the hypothesis is as follows:

**Hypothesis** **3** **(H3).** *There is a significant positive correlation between pre-use trust and perceived value in older adults’ participation in senior meal halls.*

### 2.3. Perceived Service Quality (PQ)

Perceived service quality is the consumers’ overall evaluation of the perceived service, and whether it meets their expectations [34]. The evaluation standard of the service quality is dynamic, which varies according to different service items [35], and is usually classified as interaction quality, physical service environment, and outcome quality [36]. Consumers’ previous experience can be an effective reference for perception and service quality analysis [37]. In other words, consumers must have experienced relevant services before having the perceived service quality, which will affect subsequent perception, such as satisfaction or brand loyalty [38], and further decisions about whether to use it again or not. Meanwhile, in the same way that perception is generated after using or experiencing a product or service, the perceived service quality will be affected by the previous factors of pre-use trust, perceived risk, and perceived value. In this study, perceived service quality represented the perceived service quality of the community senior meal halls participated by older adults. Therefore, the hypotheses are as follows:

**Hypothesis** **4** **(H4).** *There is a significant negative correlation between perceived risk and perceived service quality in older adults’ participation in senior meal halls.*

**Hypothesis** **5** **(H5).** *There is a significant positive correlation between perceived value and perceived service quality in older adults’ participation in senior meal halls.*

**Hypothesis** **6** **(H6).** *There is a significant positive correlation between pre-use trust and perceived service quality in older adults’ participation in senior meal halls.*

### 2.4. Perceived Satisfaction (PS)

Perceived satisfaction is seen as an evaluation by comparing expectations and actual experience of products or services [39]. The service and experience evaluation results are considered to be the main predictors of behavioral intention [40]. Perceived service quality and satisfaction can be converted, as both are consumers’ evaluation factors or opinions on particular products or services [34]. The difference between the two factors is that the judgment criteria of perceived service quality are more realistic and concrete, while satisfaction depends more on personal tendency and emotion [41]. Therefore, older adults’ satisfaction with senior meal halls represents the relative relationship before and after the participation in community senior meal halls, which reflects the internal feeling of the older adults involved. Therefore, the hypothesis is as follows:

**Hypothesis** **7** **(H7).** *There is a significant positive correlation between perceived service quality and perceived satisfaction in older adults’ participation in senior meal halls.*

### 2.5. Continuance Intentiion (CI)

Continuance intention, one of the means to measure whether products or services are successful [42], refers to the behavior shown after using or experiencing [43]. To promote the senior meal halls and make them widely accepted, in addition to the initial participation, continuance intention or behavior of the older adults is important [44]. As mentioned above, satisfaction is one of the main predictors [40,45] that affect the post-use trust [46] and continuance intention through trust [47]. In this study, continuance intention represented that, after participating in community senior meal halls, the older adults’ perceived probability of continuance use in the future from a subjective perspective. Therefore, the hypotheses are as follows:

**Hypothesis** **8** **(H8).** *There is a significant positive correlation between perceived satisfaction and post-use trust in older adults’ participation in senior meal halls.*

**Hypothesis** **9** **(H9).** *There is a significant positive correlation between perceived satisfaction and continuance intention in older adults’ participation in senior meal halls.*

**Hypothesis** **10** **(H10).** *There is a significant positive correlation between post-use trust and continuance intention in older adults’ participation in senior meal halls.*

### 2.6. Proposed Theoretical Model

**Figure 3:**
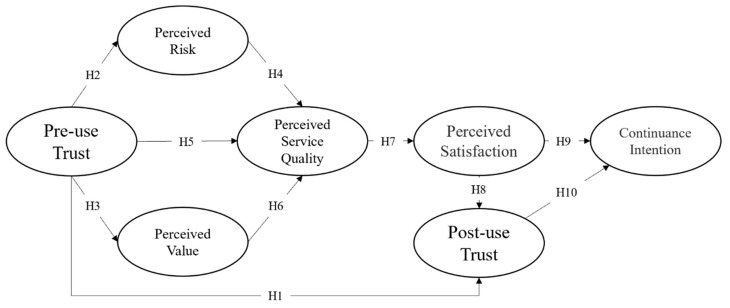
A model for continuance intention in congregate meal halls.

Based on the discussions above, ten research hypotheses and the following model (e.g., Figure 3) were proposed. In particular, the model includes seven dimensions, namely pre-use trust, perceived risk, perceived value, perceived service quality, perceived satisfaction, post-use trust, and continuance intention. 

### 2.7. Definition and Measure of Variables

Based on the research topic and related literature, this questionnaire for this study was designed by selecting items from previous studies. The variables, operationalized definitions, and source of the items are shown in Table 1.

## 3. Data and Methodology

### 3.1. Sample and Data Collection

Taking older adults who have participated in senior meal halls as subjects, from March to May 2021, the questionnaires of this study were distributed to older adults in 88 senior meal halls in Yunlin County in written form. Ethical approval for this study was obtained from the National Cheng Kung University Human Research Ethics Committee (110-417-2). All participants originally gave informed consent to participate in this survey, including consent for their data to be used for research purposes. There were 20 to 30 older adults in each hall on average, and five to six people were randomly selected. In addition to demographic variables, the Likert 7-point scale was used for the items, ranging from 1 point (Strongly disagree) to 7 points (Strongly agree). Finally, 440 samples were collected. After eliminating invalid samples (logic error or too many similar options), there were 416 valid questionnaires. The estimated parameters of this research questionnaire include 38 questions, while all 416 questionnaires met Jackson’s standard, where the ratio of estimated parameters to sample number should be higher than 1:10 [55]. Therefore, the following data analysis is based on these valid questionnaires.

Overall, among these respondents, 46.9% were male, and 53.1% were female. Over half of the participants(60.6%) had received an elementary school education. 67.1% were lived with family members, whereas 32.9% were lived independently. Most participants reported living in rural areas (62.5%). Approximately 38% of older adults reported that their main mode of transportation was using a private vehicle (motorcycle or car), 26.2% by bicycle, and 35.8% by foot. Slightly more than half of the respondents (51%) reported preparing food materials on their own, and 34% older adults received food prepared by family members, and others by neighborhood, friends, and caregivers (15%). Furthermore, older adult respondents reported “occasionally (48.1%)” for their weekly cooking frequency, followed by “frequently (21.1%)”, and “always (14.9%)”. 

### 3.2. Reliability Analysis

Reliability tests and item analyses were carried out based on the results of the pre-test questionnaire to make the research results more accurate. The unstable items were deleted to ensure the reliability and avoid discrimination of the scales. As indicated in Table 2, the Cronbach`s α value of all dimensions is greater than 0.7, meaning that all the dimensions have high reliability. Furthermore, the Cronbach’s α value of each dimension will be lower than the current results when any of the items is deleted, suggesting that no item should be deleted. In general, the scales have high reliability for further analysis.

### 3.3. Descriptive Analysis and Normal Distribution Test

The hypothesis model of this study includes seven dimensions, namely pre-use trust, perceived risk, perceived value, perceived service quality, perceived satisfaction, post-use trust, and continuance intention. Moreover, the 38 items, along with the mean and standard deviation of the samples are shown in Table 3. At the same time, Table 4 shows the normal distribution of the sample data. The absolute value of skewness of all components is less than 3, while the absolute value of kurtosis is less than 7. Thus, it is considered that the data in this study conform with the normal distribution [56].

### 3.4. Confirmatory Factor Analysis

#### 3.4.1. Convergent Validity

AMOS v22.0 software was adopted to analyze the structural equation model in this study. Through a large amount of research, AMOS was proven to be a reliable structural equation model software. In addition, the data analysis includes two stages, according to the study of Anderson and Gerbing [57]. The first stage is the measurement model, where the maximum likelihood estimation method is used to estimate parameters, including factor loading, reliability, convergent validity, and discriminant validity. Congruent with the studies on convergence validity by Hair et al. [58], Nunnally and Bernstein [59] and Fornell and Larcker [60], and the study on loading of standardized factors by Chin [61] and Hooper et al. [62], the standardized factor loading in this study is higher than 0.6, the reliability of study dimension composition is higher than 0.7, and the average variance extraction (AVE) is higher than 0.5. These results indicate that the dimensions have good convergence validity [58]. The above numbers are listed in Table 4.

For discriminant validity, according to the study of Fornell and Larcker [60], if the square root of the AVE of each dimension is greater than the correlation coefficient between dimensions, it indicates that the model has discriminant validity. Meanwhile, this study showed that the values of all diagonal lines are greater than those outside the diagonal lines, indicating that every dimension in this study has a good discriminant validity (e.g., Table 5).

#### 3.4.2. Model Fit Test

According to Jackson et al. [63], Kline [56], Schumacker [64], and Hu and Bentler [65], several indicators (MLχ^2^, DF, χ^2^/DF, RMSEA, SRMR, TLI, CFI, NFI, GFI, PGFI, PNFI, and IFI) should be selected to evaluate the structural model fit. Based on the research hypothesis and models, as shown in Table 6, most of the standard model fit evaluation indicators meet the recommended fit’s independent and combination rules. Hence, the structural model has a good fit. 

### 3.5. Path Analysis

The path analysis results of Table 7 indicate that Pre-use Trust(PT) significantly influences Post-use Trust(PO)(b = 0.296, *p* < 0.001), Perceived Risk(PR) (b = −0.226, *p* = 0.001), and PV (b = 0.469, *p* < 0.001). Perceived Risk (PR) (b = −0.165, *p* < 0.001) and Pre-use Trust(PT) (b = 0.306 *p* < 0.001) significantly influence Perceived Service Quality(PQ). Perceived Service Quality (PQ) (b = 0.360, *p* < 0.001) significantly influences Perceived Satisfaction (PS). Perceived Satisfaction (PS) (b = 0.175, *p* = 0.001) significantly influences Post-use Trust (PO). Perceived Satisfaction (PS) (b = 0.127, *p* = 0.021) and Post-use Trust(PO) (b = 0.307, *p* < 0.001) significantly influence Continuance Intention (CI).

### 3.6. Hypothesis Explanation

This study used the structural equation model to determine the influence factors of continuance intention on older adults’ participation in senior meal halls.

**Figure 4:**
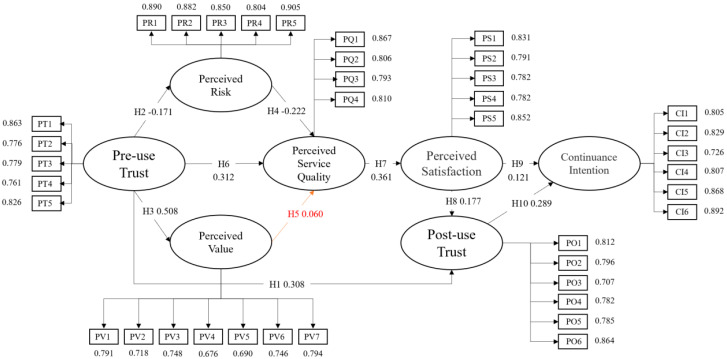
Research structure pattern diagram.

Table 7 shows the normalization coefficient of the Structural equation modeling (SEM) model in this study. The higher coefficient implies that the independent variable plays a significant role in the dependent variable. Meanwhile, Figure 4 shows the influence between variables in the structural model.

## 4. Discussion

This study identified critical factors affecting older adults’ continuous participation in senior meal halls through structural equation modeling. Our study results in several important findings. 

First, this result provides evidence that older adults’ pre-use trust has a strong positive effect on perceived value. The hypothesis from extended valence framework along with the hypothesized path from pre-use trust to usage in the perceived value stage of the model is fully supported. Further, the path coefficient is the highest, suggesting that the more positive pre-use trust the older adults hold, the more value they can feel from the senior meal halls. Through relevant government policy advocacies, shared peer and community recommendations, trial and guide promotion meetings, such information on the space equipment, food concepts, and functions of senior meal halls, older adults have already known the content of attending the meal program. It has generated public reputation on its effectiveness, and constructed the participants’ pre-use trust in senior meal halls. Moreover, after their actual participation in senior meal halls, the older adults feel motivated to enter the senior meal halls, and participate in the well-designed food promotion activities and catering services. In addition, they can get to know, talk, and share life trivia with other older adults and enthusiastic volunteers to achieve the effects on physical and mental health. Consequently, the trust level of senior meal hall participants will positively correlate with the perceived value.

Among the survey samples, 67% of older adults live with their family members. In addition to their own efforts, they need the assistance of family members or caregivers to prepare their meals. Although 84% of the older adults have cooking habits, they easily fail to have a balanced diet [9], because of their children’s eating out, a small number of diners, and an unstable meal preparation. Therefore, it is necessary for older adults to eat in a community society. Older adults of the same age may relieve their psychological pressure, and find a way to communicate with others through the interaction of eating together [66]. In addition, the correct dietary concept could be communicated in the process through the transmission and exchange of nutritional information. These findings are consistent with the results of previous studies [67,68]. Furthermore, the older adults feel at ease to eat at a fixed meal time, feeling that it can reduce the burden and trouble of their children in the meal preparation [69], and that they need not rely on relatives and friends to live. Ultimately, they feel the value of participation in senior meal halls.

Second, the results indicate pre-use trust has a strong negative effect on perceived risk. Perceived risks existed due to the limitations of individual economic ability, food preparation ability, mobility, and physical and mental degradation to older adults’ participation in senior meal halls [70,71]. The results of this study suggest that more than 60% of the older adults get to and from the senior meal halls by cars and motorcycles, while 40% walk, which may lead to traffic risks [72,73]. In addition, due to the recent improvement of dietary quality and concept, the concern for food security has also increased. Moreover, with the help of nutritionists in planning the menu, there is no need to eat leftovers several times, or excessively pickled food as in the past, as an imbalanced diet may lead to imbalanced nutrition [74]. Therefore, the risk factors such as cost standard, taste, nutritional balance, and food security are relatively reduced, proving a significant negative correlation between the degree of pre-use trust and the perceived risk. Moreover, the higher the pre-use trust is, the lower the degree of perceived risk older adults hold.

**Figure 5:**
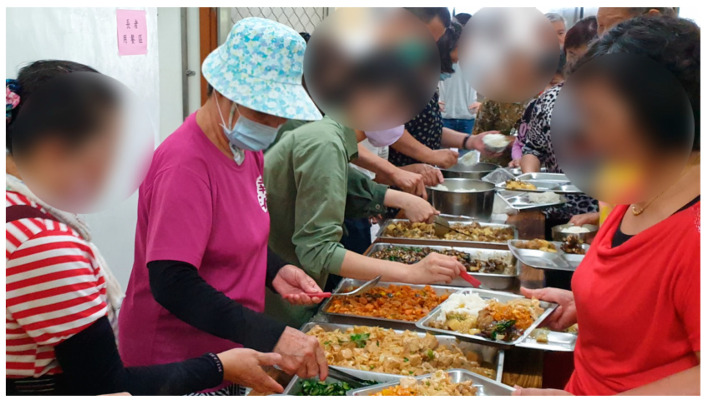
Older adults engage as volunteers for food service. (The image was taken by the author).

Third, we find that there is a significant negative correlation between the above-mentioned older adults’ perceived risk and the senior consumers’ perceived service quality. In addition, the older adults’ pre-use trust in senior meal halls and their perceived service quality has a significant positive correlation. This represents the concept of pre-use trust fully established by senior meal halls through government policies and community word-of-mouth. Currently, senior meal halls in Taiwan also encourage older adults not only to be passive recipients of care services, but also to become service providers (Figure 5). Therefore, many volunteers are involved in various service projects of the senior meal halls. The image they have established also promotes the perceived service quality of the older adults’ participation [75].

However, there is no significant correlation between the perceived value and the perceived service quality in older adults’ participation in senior meal halls. This result is different from the proposed hypothesis (H5). The reason why the perceived value does not influence perceived service quality may be that the perceived value of older adults belongs to the spiritual and psychological level of services, such as atmosphere [76], interaction mode, and diet education. Meanwhile, the perceived service quality corresponds with clear service contents (taste, space construction, charging price, and others). Older adults may pay more attention to the actual perceived service quality, while less attention is paid to the influence of psychological value. 

In terms of the overall perceived service quality, the current senior meal halls, in combination with the Long-term Care Plan of Taiwan 2.0, shall provide nutritional assessment and interview and nutritional consultation for dietary guidance, alongside providing rich and varied dishes according to the needs and physical conditions of the older adults. As for management and control of service quality, a standard menu for cooking dishes should be established to ensure stable color, flavor, and taste in each dish. In daily quality control management, the service quality of internal staff should be emphasized, and workflow should be formulated to avoid unstable quality.

An irregular satisfaction survey of the meals should be conducted to serve as a basis for adjusting and improving dishes. In addition, services, such as simple blood pressure measurement, prevention of dementia, and nutrition lectures could be added. At the same time, the circular economy model is applied to increase the participation opportunities of older adults of the same age, by which to improve their participation in the perceived satisfaction of senior meal halls. The overall evaluation of the above specific service items indicates the experience and feelings of the older adults, making them feel that perceived service quality is essential for the perceived satisfaction of the senior meal halls. As one of the core services, perceived service quality is an important reference indicator for the overall high satisfaction of the senior meal halls.

Fourth, we find that there is a significant positive correlation among perceived satisfaction, post-use trust, and continuance intention of older adults participating in senior meal halls, which shows that the perceived satisfaction influences trust attitude and continuance intention. Furthermore, the higher the perceived satisfaction, the greater the influence on the post-use trust and continuance intention among older adults. Through meal planning and food security, cleaning, interpersonal interaction, price, space layout and arrangement, and other service factors such as meal serving, the sense of community is established, where post-use trust is consequently generated.

Finally, there is a significant positive correlation between post-use trust and continuance intention on older adults’ participation in senior meal halls. This suggests that the post-use trust of this study has an influence on the continuance intention behavior. Use trust will become one of the factors in considering whether older adults continue to participate in the senior meal hall plan. The higher the trust, the more concerned they will be about hall equipment, space cooperation, and dining atmosphere. Consequently, a higher continuance intention of participation is generated [65]. In contrast, continuous participation in senior meal halls gradually changes the diet and lifestyle of older adults and promotes successful aging.

## 5. Conclusions and Suggestions

### 5.1. Conclusions

With the rapid development of the aging society, the older adults’ catering services and lifestyles are constantly changing. In Taiwan, by eating together in the form of senior meal halls run by communities and relying on stable government planning and mechanism, it is expected that the dietary needs of the older adults could be met, and successful and healthy aging could be achieved. Previous studies on the senior meal halls for older adults are mostly qualitative analyses, lacking empirical data. Based on various important theories, the contribution of this study is to take pre-use trust as the first level of influence source of post-use trust, perceived risk, and perceived value, and establish a theoretical framework to influence the initial attitude of older adults participating in senior meal halls. The structural equation model was used to determine the main factors affecting the perceived service quality, perceived satisfaction, and continuance intention. Moreover, through an analysis of the related influence of this study, most of the dimensions have a direct or indirect influence on the continuance intention of participating in senior meal halls. The model of influencing factors on continuance intention of older adults’ participation in senior meal halls established in this study is an acceptable model, which suggests that this model has a certain effect on explaining the intention of the older adults’ participation in senior meal halls. Consequently, this can also provide an important reference for future policy and industrial development.

In addition, the results also show that the perceived service quality is the main factor that predicts the perceived satisfaction, while the perceived satisfaction of the older adults plays an important role in this survey. It shows that if the older adults are satisfied with the service quality (such as food flavor, space environment, atmosphere, among other factors) provided by the senior meal halls, which will accordingly affect the post-use trust, they will show a positive continuance intention to participate in the senior meal halls. 

Through this survey, we believe that our study provided comprehensive insights and evidence into older adult’s continuance intention process in congregate meal halls. The survey also indicated that, in addition to their concern about the food provided, the older adults also pay attention to how they can avail of health education publication services, nutrition consulting services, welfare information, or recommendation services through senior meal halls [77]. Therefore, it is essential to determine how to develop other regular activities to increase interaction frequency, social interaction, and willingness to continue participating [78]. This may include increasing the participation of older adults by recommendation, front-end tasks such as picking vegetables, washing vegetables, or menu planning, and follow-up tasks such as serving dishes, placing dishes, tidying up, or space arrangement, so that older adults can have a sense of achievement in social participation and labor efforts. At the same time, efforts should be made to improve the mobility limitations of older adults due to aging or inconvenient transportation. All of these will be indispensable factors for older adults to continue participating in senior meal halls. 

### 5.2. Research Limitations and Future Research Suggestions

Some limitations of this study may indicate future research directions.

Firstly, this study explored the mutual influencing factors of the older adults’ trust attitude, perceived value, and continuance intention of participating in senior meal halls. Thus, researchers can continue to explore multiple dietary plans with different meal-sharing patterns and service scales in the future. Secondly, due to the limitation of time and resources, this study only collected questionnaires from senior meal halls run by public sectors in central Taiwan. Although a highly aged county in Taiwan was taken as a reference value, individual differences still exist in different counties and cities. Therefore, different opinions may be held on the topic of this study. It is suggested that future research explores senior meal halls run by different counties, cities, or organizations to provide a reference for the government, communities, and relevant industries.

## Figures and Tables

**Table 1:** Definition of variable operability and reference scales.

Research Variable	Operability Definition	Reference Scale
Pre-use trust	Trust level before individuals participate in community senior meal halls	[16,48,49]
Perceived risk	Expected perceived risk probability of individuals who participate in community senior meal halls	[50]
Perceived value	Perceived positive results probability of individuals participate in community senior meal halls	[51]
Perceived service quality	Perceived service quality level of individuals who participate in community senior meal halls	[52]
Perceived satisfaction	Relative relationship between experience before and after individuals participate in community senior meal halls	[19,48,53]
Post-use trust	Trust level after individuals participate in community senior meal halls	[16,48,49]
Continuance intention	Perceived probability of continuance of use in the future from a subjective perspective by individuals who participate in community senior meal halls	[54]

**Table 2:** Reliability and items analysis of the dimension items.

Dimension	Question	CITC	Cronbach’s Alpha Value after Deleting the Item	Cronbach’s Alpha Value
Pre-use trust	PT1	0.801	0.867	0.900
	PT2	0.731	0.883	
	PT3	0.735	0.882	
	PT4	0.720	0.885	
	PT5	0.772	0.874	
perceived risk	PR1	0.850	0.920	0.937
	PR2	0.848	0.920	
	PR3	0.820	0.925	
	PR4	0.775	0.933	
	PR5	0.871	0.916	
Perceived value	PV1	0.739	0.871	0.893
	PV2	0.675	0.879	
	PV3	0.692	0.877	
	PV4	0.631	0.884	
	PV5	0.653	0.881	
	PV6	0.698	0.876	
	PV7	0.747	0.870	
Perceived service quality	PQ1	0.790	0.850	0.892
	PQ2	0.749	0.865	
	PQ3	0.749	0.866	
	PQ4	0.760	0.862	
Perceived satisfaction	PS1	0.787	0.877	0.904
	PS2	0.741	0.887	
	PS3	0.734	0.888	
	PS4	0.740	0.887	
	PS5	0.795	0.875	
Post-use trust	PO1	0.769	0.893	0.911
	PO2	0.752	0.895	
	PO3	0.687	0.904	
	PO4	0.741	0.896	
	PO5	0.751	0.895	
	PO6	0.810	0.887	
Continuance intention	CI1	0.761	0.915	0.925
	CI2	0.779	0.912	
	CI3	0.697	0.923	
	CI4	0.778	0.913	
	CI5	0.836	0.905	
	CI6	0.857	0.902	

Note: CITC:corrected item-total correlation.

**Table 3:** Descriptive analysis and normal distribution test of dimension items.

	Number	Average Value	SD	Skewness	Kurtosis
Pre-use trust	416	5.10	1.13	−1.39	2.31
Perceived risk	416	4.20	1.52	−0.47	−1.04
Perceived value	416	4.72	1.10	−0.86	0.55
Perceived service quality	416	5.24	1.16	−0.77	0.46
Perceived satisfaction	416	5.09	1.17	−0.59	−0.67
Post-use trust	416	4.90	1.16	−0.61	−0.27
Continuance intention	416	5.12	1.24	−1.08	0.77

Note: SD: Standard Deviation

**Table 4:** Measurement model.

Dimension	Item	Unstd.	S.E.	Unstd./S.E.	*p*-Value	Std.	CR	AVE
PT	PT1	1.000	-	-	-	0.867	0.900	0.644
	PT2	0.839	0.045	18.834	0.000	0.774		
	PT3	0.826	0.044	18.926	0.000	0.777		
	PT4	0.826	0.045	18.397	0.000	0.762		
	PT5	0.892	0.043	20.909	0.000	0.828		
PR	PR1	1.000	-	-	-	0.890	0.938	0.752
	PR2	0.920	0.035	26.180	0.000	0.883		
	PR3	0.902	0.037	24.196	0.000	0.850		
	PR4	0.822	0.038	21.710	0.000	0.804		
	PR5	0.961	0.035	27.687	0.000	0.905		
PV	PV1	1.000	-	-	-	0.793	0.894	0.546
	PV2	0.888	0.057	15.462	0.000	0.721		
	PV3	0.920	0.057	16.039	0.000	0.743		
	PV4	0.899	0.063	14.340	0.000	0.677		
	PV5	0.870	0.059	14.726	0.000	0.693		
	PV6	0.921	0.057	16.105	0.000	0.746		
	PV7	0.981	0.057	17.295	0.000	0.790		
PQ	PQ1	1.000	-	-	-	0.861	0.892	0.674
	PQ2	0.914	0.046	19.765	0.000	0.811		
	PQ3	0.859	0.045	19.254	0.000	0.797		
	PQ4	0.931	0.047	19.904	0.000	0.815		
PS	PS1	1.000	-	-	-	0.834	0.904	0.655
	PS2	0.896	0.048	18.557	0.000	0.791		
	PS3	0.890	0.049	18.263	0.000	0.782		
	PS4	0.909	0.049	18.363	0.000	0.785		
	PS5	1.000	0.049	20.559	0.000	0.849		
PO	PO1	1.000	-	-	-	0.819	0.911	0.632
	PO2	0.966	0.052	18.568	0.000	0.796		
	PO3	0.813	0.051	16.007	0.000	0.713		
	PO4	0.905	0.050	18.223	0.000	0.785		
	PO5	0.936	0.052	18.140	0.000	0.783		
	PO6	0.997	0.048	20.956	0.000	0.867		
CI	CI1	1.000	-	-	-	0.805	0.926	0.678
	CI2	0.958	0.049	19.540	0.000	0.830		
	CI3	0.785	0.048	16.325	0.000	0.726		
	CI4	0.931	0.049	18.843	0.000	0.809		
	CI5	1.009	0.049	20.800	0.000	0.867		
	CI6	1.066	0.049	21.702	0.000	0.893		

Note: Unstd.: Unstandardized factor loadings, S.E.: Standard Error, Std.: Standardized factor loadings, CR: Composite Reliability, AVE: Average Variance Extracted.

**Table 5:** Discriminant validity for the measurement model.

	PT	PR	PV	PQ	PS	PO	CI
PT	0.802						
PR	−0.151	0.867					
PV	0.452	−0.228	0.738				
PQ	0.315	−0.262	0.220	0.820			
PS	0.303	−0.173	0.431	0.290	0.809		
PO	0.301	−0.160	0.319	0.500	0.242	0.794	
CI	0.316	−0.176	0.254	0.398	0.164	0.287	0.823

Note: The items on the diagonal in bold represent the square roots of the Average Variance Extracted(AVE); off-diagonal elements are the correlation estimates.

**Table 6:** Evaluation results.

Indicators	Norm	Results	Judgment
MLχ^2^	The small the better	1349.508	
DF	The large the better	655.000	
χ^2^/DF	1 < χ^2^/DF < 5	2.060	Yes
RMSEA	<0.08	0.051	Yes
SRMR	<0.08	0.036	Yes
TLI (NNFI)	>0.9	0.931	Yes
CFI	>0.9	0.936	Yes
NFI	>0.9	0.883	No
GFI	>0.8	0.858	Yes
PGFI	>0.5	0.759	Yes
PNFI	>0.5	0.823	Yes
IFI	>0.9	0.936	Yes

Note: MLχ^2^: ML chi-square, DF: Degrees of Freedom, χ^2^/DF: normed Chi-square, RMSEA: Root Mean Square Error Approximation, SRMR: Standardized Root Mean Square Residual, TLI: Tucker-Lewis Index, CFI: Comparative Fit Index, NFI: Normative Fit Index, GFI: Goodness of Fit index, PGFI: Parsimony Goodness of Fit Index, PNFI: Parsimony Normed Fit Index, IFI: Incremental Fit Index.

**Table 7:** Regression coefficient.

Hypothesis	DV	IV	Unstd	S.E.	Unstd./S.E.	*p*-Value	Std.	Results
H1	PO	PT	0.296	0.051	5.823	0.000	0.308	Yes
H2	PR	PT	−0.226	0.070	−3.251	0.001	−0.171	Yes
H3	PV	PT	0.469	0.050	9.451	0.000	0.508	Yes
H4	PQ	PR	−0.165	0.037	−4.413	0.000	−0.222	Yes
H5	PQ	PV	0.063	0.064	0.992	0.321	0.060	No
H6	PQ	PT	0.306	0.061	5.052	0.000	0.312	Yes
H7	PS	PQ	0.360	0.053	6.738	0.000	0.361	Yes
H8	PO	PS	0.175	0.051	3.426	0.001	0.177	Yes
H9	CI	PS	0.127	0.055	2.306	0.021	0.121	Yes
H10	CI	PO	0.307	0.057	5.369	0.000	0.289	Yes

Note: DV: Dependent Variable, IV: Independent Variable

## Data Availability

The data presented in this study are available on request from the first author.
